# 2,3,7,8-Tetrachlorodibenzo-*p*-dioxin (TCDD)-elicited effects on bile acid homeostasis: Alterations in biosynthesis, enterohepatic circulation, and microbial metabolism

**DOI:** 10.1038/s41598-017-05656-8

**Published:** 2017-07-19

**Authors:** Kelly A. Fader, Rance Nault, Chen Zhang, Kazuyoshi Kumagai, Jack R. Harkema, Timothy R. Zacharewski

**Affiliations:** 1 Department of Biochemistry & Molecular Biology, Michigan State University, East Lansing, MI 48824 USA; 2 Institute for Integrative Toxicology, Michigan State University, East Lansing, MI 48824 USA; 3 Department of Chemistry, Michigan State University, East Lansing, MI 48824 USA; 4 Department of Pathobiology & Diagnostic Investigation, Michigan State University, East Lansing, MI 48824 USA

## Abstract

2,3,7,8-tetrachlorodibenzo-*p*-dioxin (TCDD) is a persistent environmental contaminant which elicits hepatotoxicity through activation of the aryl hydrocarbon receptor (AhR). Male C57BL/6 mice orally gavaged with TCDD (0.01–30 µg/kg) every 4 days for 28 days exhibited bile duct proliferation and pericholangitis. Mass spectrometry analysis detected a 4.6-fold increase in total hepatic bile acid levels, despite the coordinated repression of genes involved in cholesterol and primary bile acid biosynthesis including *Cyp7a1*. Specifically, TCDD elicited a >200-fold increase in taurolithocholic acid (TLCA), a potent G protein-coupled bile acid receptor 1 (GPBAR1) agonist associated with bile duct proliferation. Increased levels of microbial bile acid metabolism loci (*bsh*, *baiCD*) are consistent with accumulation of TLCA and other secondary bile acids. Fecal bile acids decreased 2.8-fold, suggesting enhanced intestinal reabsorption due to induction of ileal transporters (*Slc10a2*, *Slc51a*) and increases in whole gut transit time and intestinal permeability. Moreover, serum bile acids were increased 45.4-fold, consistent with blood-to-hepatocyte transporter repression (*Slco1a1*, *Slc10a1*, *Slco2b1*, *Slco1b2*, *Slco1a4*) and hepatocyte-to-blood transporter induction (*Abcc4*, *Abcc3*). These results suggest that systemic alterations in enterohepatic circulation, as well as host and microbiota bile acid metabolism, favor bile acid accumulation that contributes to AhR-mediated hepatotoxicity.

## Introduction

Metabolic syndrome (MetS) is defined as a collection of cardiometabolic factors including obesity, dyslipidemia, hypertension, and hyperglycemia which increase the risk of developing cardiovascular disease, type II diabetes, and hepatocellular carcinoma^1^. The prevalence of MetS among adults in the United States is ~30%, representing an emerging epidemic of concern as the population ages^2^. In the liver, MetS can manifest as non-alcoholic fatty liver disease (NAFLD) where hepatic steatosis (lipid accumulation) progresses to steatohepatitis (steatosis with inflammation) with fibrosis (collagen deposition)^3^. High fat diet, sedentary behavior, and various genetic loci are key risk factors for MetS and NAFLD development. However, accumulating evidence suggests that exposure to 2,3,7,8-tetrachlorodibenzo-*p*-dioxin (TCDD) and other environmental contaminants play an underappreciated role^4–6^.

TCDD is the prototypical ligand for a structurally diverse group of synthetic chemicals, natural products, and endogenous metabolites that activate the aryl hydrocarbon receptor (AhR)^7^. Ligand binding initiates the dissociation of chaperone proteins, triggering translocation of the cytoplasmic AhR to the nucleus and heterodimerization with the aryl hydrocarbon receptor nuclear translocator (ARNT). In the canonical pathway, the liganded AhR-ARNT complex binds to dioxin response elements (DREs) within the promoter region of target genes, leading to recruitment of transcriptional co-regulators and altered gene expression^8^. However, an increasing number of studies report DRE-independent mechanisms of differential gene expression^9, 10^. AhR-mediated gene expression changes elicit a broad spectrum of toxic responses including wasting, tumor promotion, immunosuppression, dermal lesions, teratogenicity, and hepatotoxicity in a species-, sex-, age-, tissue-, and cell-specific manner^11, 12^. In mice, TCDD-elicits AhR-dependent lipid accumulation, immune cell infiltration, and bile duct proliferation^13–15^, which progresses to steatohepatitis with fibrosis following repeated treatment^16, 17^. Beyond AhR activation, the specific target genes and altered biological processes that contribute to TCDD-elicited hepatotoxicity remain poorly understood.

Bile acids, the predominant organic solutes in bile, are synthesized from cholesterol in the liver, stored in the gallbladder, and secreted into the duodenal lumen following consumption of a meal. These amphipathic molecules promote the solubilization and absorption of dietary lipids and lipid-soluble vitamins, and provide an excretion mechanism for excess hepatic cholesterol. Additionally, specific bile acid species are endogenous ligands for a number of receptors including farnesoid X receptor (FXR), G protein-coupled bile acid receptor 1 (GPBAR1), pregnane X receptor (PXR), vitamin D receptor (VDR), liver X receptor α (LXRα), and sphingosine-1-phosphate receptor 2 (S1P2)^18, 19^. Consequently, they serve as important signaling molecules that regulate not only bile and cholesterol homeostasis, but also glucose and energy metabolism. Moreover, bile acids can elicit hepatocyte and non-parenchymal cell injury by disrupting cell membranes through their detergent action and by contributing to oxidative stress through reactive oxygen species (ROS) generation^20^. Furthermore, they promote inflammation by inducing pro-inflammatory mediators such as cytokines (IL-1β, CSF1), chemokines (CXCL1, CXCL2, CCL2), and adhesion molecules (VCAM-1)^21^. The dysregulation of mitochondrial function, depletion of ATP, and activation of signaling cascades can trigger bile acid-induced necrosis^20^. Therefore, bile acid accumulation and/or disrupted biliary flow (cholestasis) can contribute to hepatotoxicity and impaired liver function, while promoting the development of fibrosis.

Previous studies have reported that TCDD and related AhR ligands increase bile acid levels in serum^22, 23^, decrease biliary flow^24^, and induce bile duct epithelial cell proliferation^25^. TCDD also alters hepatic expression of genes associated with cholesterol metabolism and bile acid biosynthesis^26^, and exacerbates cholestatic liver disease induced by bile duct ligation (BDL)^27^. To further investigate AhR-mediated qualitative and quantitative changes in bile acid profiles, liquid chromatography mass spectrometry (LC-MS) was integrated with hepatic and ileal RNA-Seq analyses, along with complementary histopathological assessments. The effects of TCDD on intestinal permeability and motility, and gut microbiota bile acid metabolism were also examined. Despite the coordinated repression of genes involved in cholesterol and primary bile acid biosynthesis, total bile acid levels increased in the liver and serum, while fecal levels decreased. In particular, TCDD increased levels of hepatic taurolithocholic acid (TLCA), a potent GPBAR1 agonist associated with bile duct proliferation and cholestasis^28, 29^. Overall, TCDD-elicited differential expression of bile acid transporters, increased intestinal permeability, decreased gut motility, and alterations in the gut microbiome suggest disruption of enterohepatic circulation and bile acid metabolism. Collectively, the qualitative and quantitative changes in bile acid homeostasis are consistent with alterations in host and intestinal microbiota metabolism, yielding hepatotoxic species that contribute to the development of steatohepatitis with fibrosis.

## Results

### Histopathology

**Figure 1:**
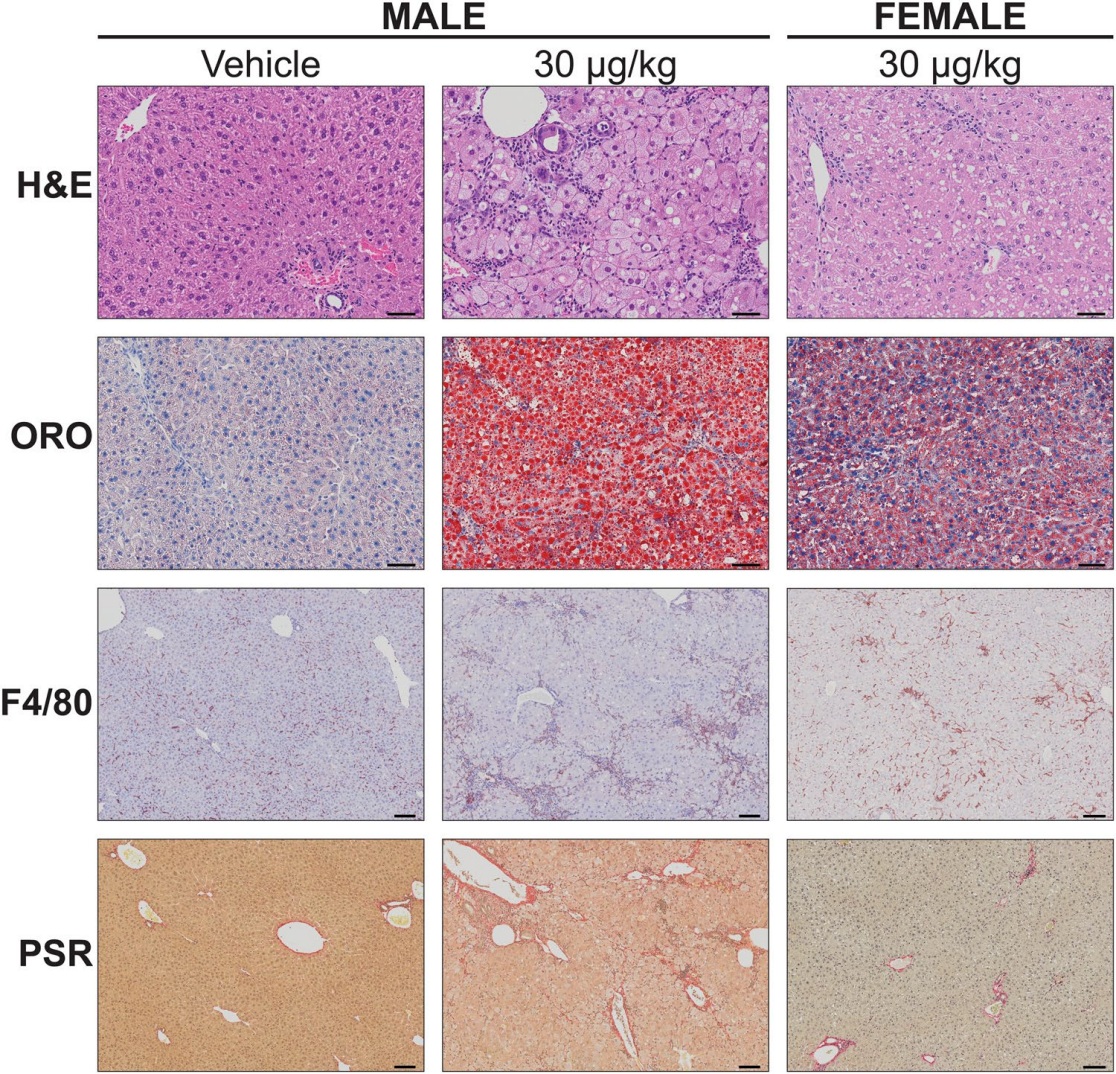
Histological evaluation of livers from male and female C57BL/6 mice orally gavaged with sesame oil vehicle or 30 µg/kg TCDD every 4 days for 28 days. Livers were stained with hematoxylin and eosin (H&E) for general assessment, Oil Red O (ORO) for neutral lipids, F4/80 for macrophages, and Picrosirius Red (PSR) for collagen deposition. Scale bars represent 50 µm for H&E and ORO, and 100 µm for F4/80 and PSR.

**Figure 2:**
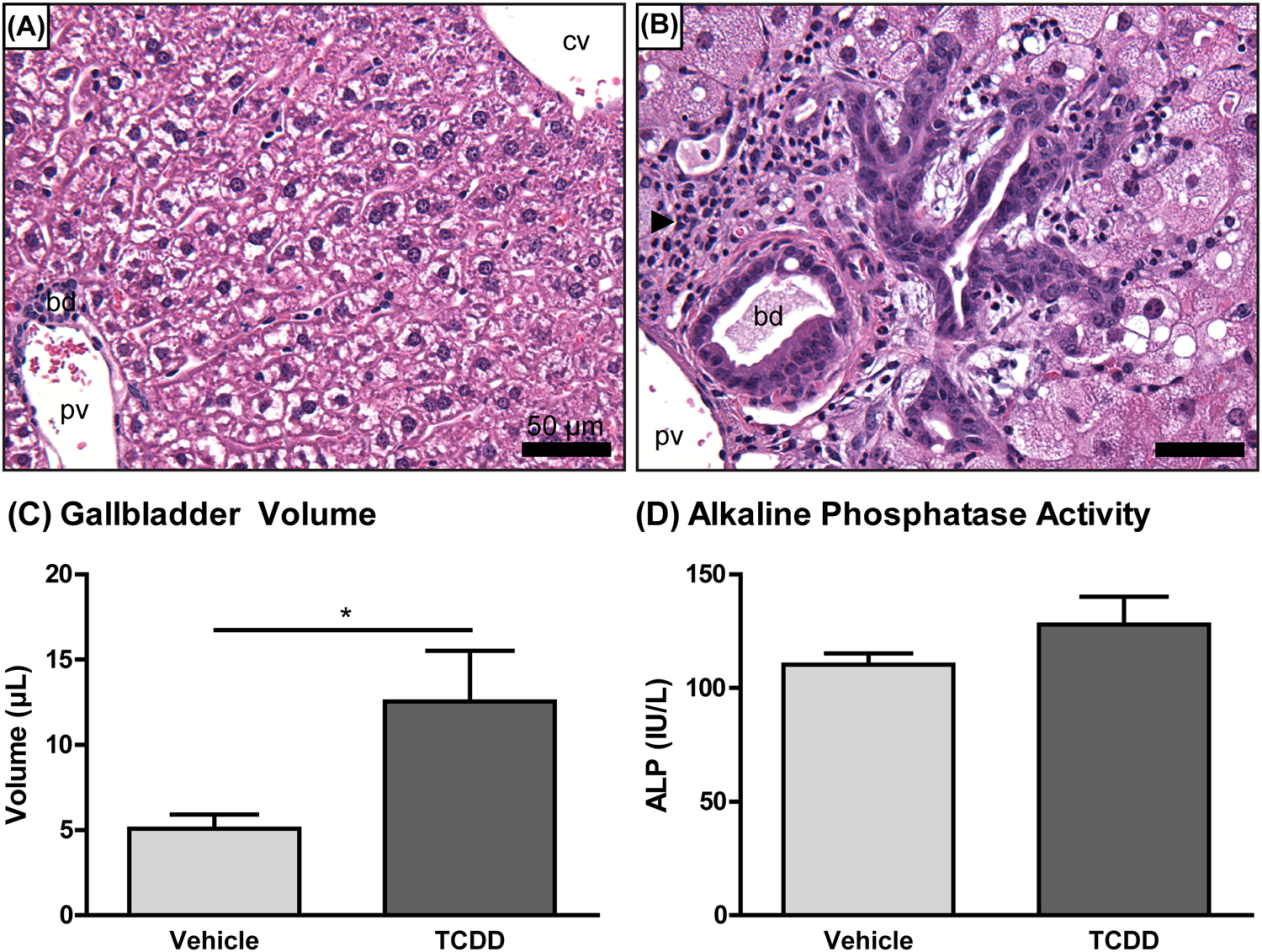
Representative photomicrographs of hematoxylin and eosin-stained livers from male C57BL/6 mice orally gavaged with (**A**) sesame oil vehicle or (**B**) 30 µg/kg TCDD every 4 days for 28 days. Bile duct proliferation and pericholangitis (arrow) were identified at 30 µg/kg TCDD. cv = central vein; pv = portal vein; bd = bile duct; bar = 50 µm. (**C**) Gallbladder volume and (**D**) serum alkaline phosphatase (ALP) activity of male C57BL/6 mice orally gavaged with sesame oil vehicle or 30 µg/kg TCDD every 4 days for 28 days. Bars represent the average ± standard error of the mean for at least 5 biological replicates. Statistical significance (*p ≤ 0.05) was determined using a Student’s t-test performed in SAS 9.3.

Previous studies report hepatic steatosis, immune cell infiltration, fibrosis, and bile duct proliferation in male mice following treatment with TCDD and related AhR agonists^14, 17, 25, 30, 31^. Comparable effects are reported in female C57BL/6 mice orally gavaged with TCDD every 4 days (d) for either 28 or 92d^16, 32^. Here, the histopathological features associated with TCDD-elicited hepatotoxicity in both female and male C57BL/6 mice orally gavaged with TCDD every 4d for 28d were compared. Histopathological scoring suggests the incidence and severity of vacuolization, inflammatory cell infiltration, bile duct proliferation, and periportal fibrosis were consistently greater in male mice compared to females (Table 1; Fig. 1). More specifically, hepatocyte vacuolization (fatty change) was first observed in males at 0.3 µg/kg and in females at 10 µg/kg TCDD. Fat accumulation was more severe in males compared to females at ≥3 µg/kg TCDD (Table 1). QuHAnT analysis of ORO-stained sections indicated that hepatic fat accumulation at 30 µg/kg TCDD was 6.2-fold greater in males compared to females (Fig. 1; Supplementary Fig. S1), consistent with the pathologist’s assessment. Minimal to slight hepatocyte necrosis and inflammatory cell infiltration (e.g. neutrophils and lymphocytes) were present in males at ≥3 µg/kg and in females at 30 µg/kg. Both were more severe in males at 30 µg/kg compared to the females (Table 1). F4/80 labeling revealed that lymphocyte foci consisted primarily of macrophages, suggesting infiltration (Fig. 1). Bile duct proliferation was observed in both sexes at 30 µg/kg, with a higher incidence in males, while inflammation surrounding the bile ducts (pericholangitis) was exclusive to males (Table 1; Fig. 2A,B). Similarly, PSR-staining revealed that the incidence of periportal fibrosis at 30 µg/kg was higher in males compared to females (Table 1; Fig. 1). In summary, male mice exhibited greater sensitivity to TCDD-elicited hepatotoxicity compared to females, and exhibited more severe histopathology at lower doses. Subsequent studies therefore focused on responses in male mice.

**Table 1:** Comparison of TCDD-elicited histopathological changes between male and female C57BL/6 mice.

**TCDD** (**µg/kg**)		**0**.**3**		**1**		**3**			**10**			**30**		
**Sex**		Male	Female	Male	Female	Male	Female		Male	Female		Male	Female	
Number of animals		8	8	8	6	8	6		8	4		8	4	
Vacuolation (centrilobular)	Mean Grade	0.1 ± 0.4	—	0.9 ± 0.8	—	1.0 ± 0.8	—	*	3.0 ± 0.5	0.5 ± 0.3	*	4.0 ± 0.0	2.0 ± 1.2	*
	Incidence	1/8	0/8	5/8	0/6	6/8	0/6		8/8	1/4		8/8	4/4	
Necrosis; Inflammatory cell foci	Mean Grade	—	—	—	—	0.3 ± 0.5	—		0.8 ± 0.5	—		1.3 ± 0.5	0.3 ± 0.5	*
	Incidence	0/8	0/8	0/8	0/6	2/8	0/6		6/8	0/4		8/8	1/4	
Bile duct proliferation	Mean Grade	—	—	—	—	—	—		—	—		0.9 ± 0.8	0.3 ± 0.5	
	Incidence	0/8	0/8	0/8	0/6	0/8	0/6		0/8	0/4		5/8	1/4	
Fibrosis (periportal)	Mean Grade	—	—	—	—	—	—		—	—		0.6 ± 0.5	0.3 ± 0.5	
	Incidence	0/8	0/8	0/8	0/6	0/8	0/6		0/8	0/4		5/8	1/4	

Histopathological grade: 0 = Within normal limits, 1 = Minimal, 2 = Slight, 3 = Moderate, 4 = Marked. No noteworthy hepatic changes were observed in mice treated with sesame oil vehicle or ≤0.1 µg/kg TCDD. Mean Grade = Mean of histopathological grades ±SD. *p ≤ 0.05 (compared between males and females at the same dose, Mann—Whitney rank sum test). Incidence = Number of mice in which the histopathological feature was present.

### Gross Morphology and Clinical Chemistry

Terminal body weights and tissue weights for the mice used in this study were previously reported^33^. Gallbladder volume increased 2.5-fold in mice treated with 30 µg/kg TCDD compared to controls (Fig. 2C). The gallbladder distention is consistent with previous studies^31^. Serum alkaline phosphatase (ALP) activity was unchanged at 30 µg/kg, suggesting TCDD did not damage bile duct epithelial cells (cholangiocytes) (Fig. 2D).

### Cholesterol and Primary Bile Acid Biosynthesis

**Figure 3:**
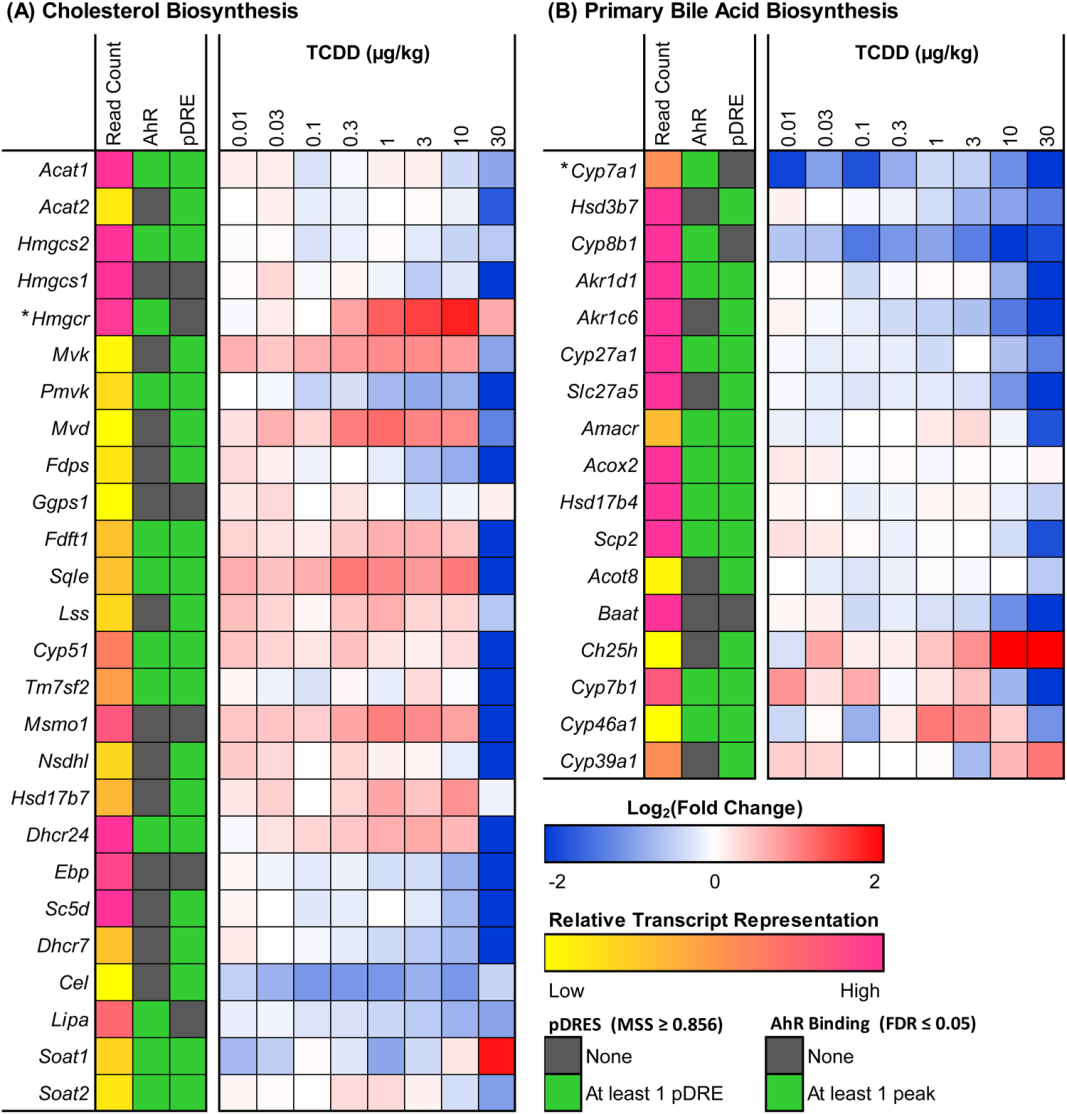
TCDD-elicited hepatic differential expression of genes involved in (**A**) cholesterol and (**B**) primary bile acid biosynthesis in male C57BL/6 mice orally gavaged with sesame oil vehicle or TCDD (0.01–30 µg/kg TCDD) every 4 days for 28 days. Color scale represents the log_2_(fold change) for differential gene expression relative to vehicle controls, as determined through RNA-Seq analysis (n = 3). The presence of pDREs (MSS ≥ 0.856) and AhR enrichment peaks (FDR ≤ 0.05) at 2 h are shown as green boxes. Read count represents the raw number of aligned reads to each transcript indicating potential level of expression, where yellow represents a lower level of expression (≤500 reads) and pink represents a higher level of expression (≥10,000). ﻿The rate-limiting enzyme of each pathway is marked with an asterisk (*).﻿

Cholesterol is not only essential for maintaining cell membrane fluidity, but it also serves as the precursor for steroid hormone and bile acid biosynthesis. In mammals, the liver is the primary site of acetyl-CoA conversion to cholesterol, while the intestine also exhibits *de novo* synthesis capabilities. Hepatic cholesterol (not confirmed by high resolution MS/MS) and cholesterol ester levels increased 9.0- and 11.3-fold, respectively, in male C57BL/6 mice orally gavaged with 30 µg/kg TCDD every 4d for 28d^34^. The committed step of cholesterol biosynthesis involves the conversion of 3-hydroxy-3-methylglutaryl-CoA to mevalonate catalyzed by the rate-limiting enzyme 3-hydroxy-3-methylglutaryl-CoA reductase (HMGCR). Hepatic expression of *Hmgcr* was dose-dependently induced at 0.3–10 µg/kg TCDD (max 3.4-fold), with no significant induction at 30 µg/kg (Fig. 3). Other enzymes involved in cholesterol biosynthesis including mevalonate kinase (*Mvk*), mevalonate diphosphate decarboxylase (*Mvd*), squalene epoxidase (*Sqle*), lanosterol synthase (*Lss*), methylsterol monoxygenase 1 (*Msmo1*), and 24-dehydrocholesterol reductase (*Dhcr24*) exhibited a comparable dose-dependent expression pattern (Fig. 3). This suggests that at low doses, AhR activation induces cholesterol biosynthesis, consistent with a 10.3-fold increase in AhR genomic binding at the *Hmgcr* loci. However, negative feedback at 30 µg/kg TCDD may limit *de novo* biosynthesis.

Several genes involved in cholesterol biosynthesis, including 3-hydroxy-3-methylglutaryl-CoA synthase 1 (*Hmgcs1*), *Hmgcr*, farnesyl diphosphate synthetase (*Fdps*), and farnesyl diphosphate farnesyl transferase 1 (*Fdft1*), are transcriptionally regulated by sterol regulatory element-binding protein 2 (SREBP2) binding to sterol response elements (SREs). Elevated intracellular cholesterol levels prevent intramembrane SREBP2 proteolysis and subsequent nuclear translocation, thereby reducing SRE-regulated gene transcription. Consequently, higher hepatic cholesterol levels may repress SREBP2-mediated transcription. Furthermore, *Srebf2* (encodes SREBP2) was repressed 2.7-fold at 30 µg/kg TCDD (Supplementary Table S1), which may further compromise expression of cholesterol biosynthetic genes.

Approximately 90% of excess cholesterol is excreted from the body by hepatic conversion to bile acids. In humans, the two primary bile acids synthesized directly by the liver are cholic acid (CA) and chenodeoxycholic acid (CDCA). Rodent livers hydroxylate CDCA at the 6β-positon yielding α-muricholic acid (α-MCA), which can epimerize at the 7-OH position to form β-muricholic acid (β-MCA), and therefore α/β-MCA are also primary bile acids in mice^35^. In the classical bile acid biosynthesis pathway, cholesterol is directly 7α-hydroxylated by CYP7A1, representing the rate-limiting step. Alternative pathways involve initial hydroxylation at C24, C25, or C27 catalyzed by CYP46A1, cholesterol 25-hydroxylase (CH25H), and CYP27A1, respectively, followed by 7α-hydroxylation. TCDD repressed *Cyp7a1* 39.7-fold with a 2.3-fold enrichment in AhR genomic binding (Fig. 3). Furthermore, 9 of the 12 downstream enzymes involved in the classical pathway were also repressed, including hydroxy-delta-5-steroid dehydrogenase 3β7 (*Hsd3b7*; 2.5-fold), *Cyp8b1* (4.4-fold), aldo-keto reductase 1D1 (*Akr1d1*; 16.3-fold), aldo-keto reductase 1C6 (*Akr1c6*; 22.1-fold), *Cyp27a1* (2.4-fold), *Slc27a5* (16.0-fold), α-methylacyl-CoA racemase (*Amacr*; 3.3-fold), sterol carrier protein 2 (*Scp2*; 3.4-fold), and bile acid-Coenzyme A: amino acid N-acyltransferase (*Baat*; 12.0-fold) (Fig. 3). With the exception of CYP8B1, which is unique to CA synthesis, these enzymes are required for synthesis of all murine primary bile acids. Interestingly, AhR binding was enriched 2.2-fold within *Cyp8b1*, suggesting AhR-mediated regulation of the CDCA to CA ratio. There are four genes unique to the alternative pathway of bile acid synthesis: *Cyp46a1* and *Cyp7b1*, which were repressed 2.1- and 7.6-fold, respectively, as well as *Cyp39a1* and *Ch25h*, which were induced 2.1- and 6.1-fold, respectively, by TCDD (Fig. 3).

Bile acid biosynthesis is primarily regulated by two negative feedback loops that repress *Cyp7a1*, both involving bile acid-mediated activation of FXR. Hepatic FXR induces short heterodimer partner (*Shp*, aka *Nr0b2*), which then antagonizes liver receptor homolog 1 (LRH-1)- and hepatocyte nuclear factor 4α (HNF4α)-mediated *Cyp7a1* expression. However, TCDD repressed hepatic *Nr0b2* 22.2-fold (Supplementary Table S1). Alternatively, activation of intestinal FXR induces ileal fibroblast growth factor 15 (*Fgf15*) expression. FGF15 binds its hepatic heterodimer receptor consisting of fibroblast growth factor receptor 4 (FGFR4) and klotho β (KLB), which causes *Cyp7a1* repression and regulates bile flow. Yet, hepatic *Fgfr4* and *Klb* were repressed 4.7- and 8.1-fold respectively, while ileal *Fgf15* was repressed 9.7-fold at 30 µg/kg TCDD (Supplementary Table S1). Therefore, it is unlikely that either feedback loop is responsible for *Cyp7a1* repression.

### Hepatic, Serum, and Fecal Bile Acid Profiles

**Figure 4:**
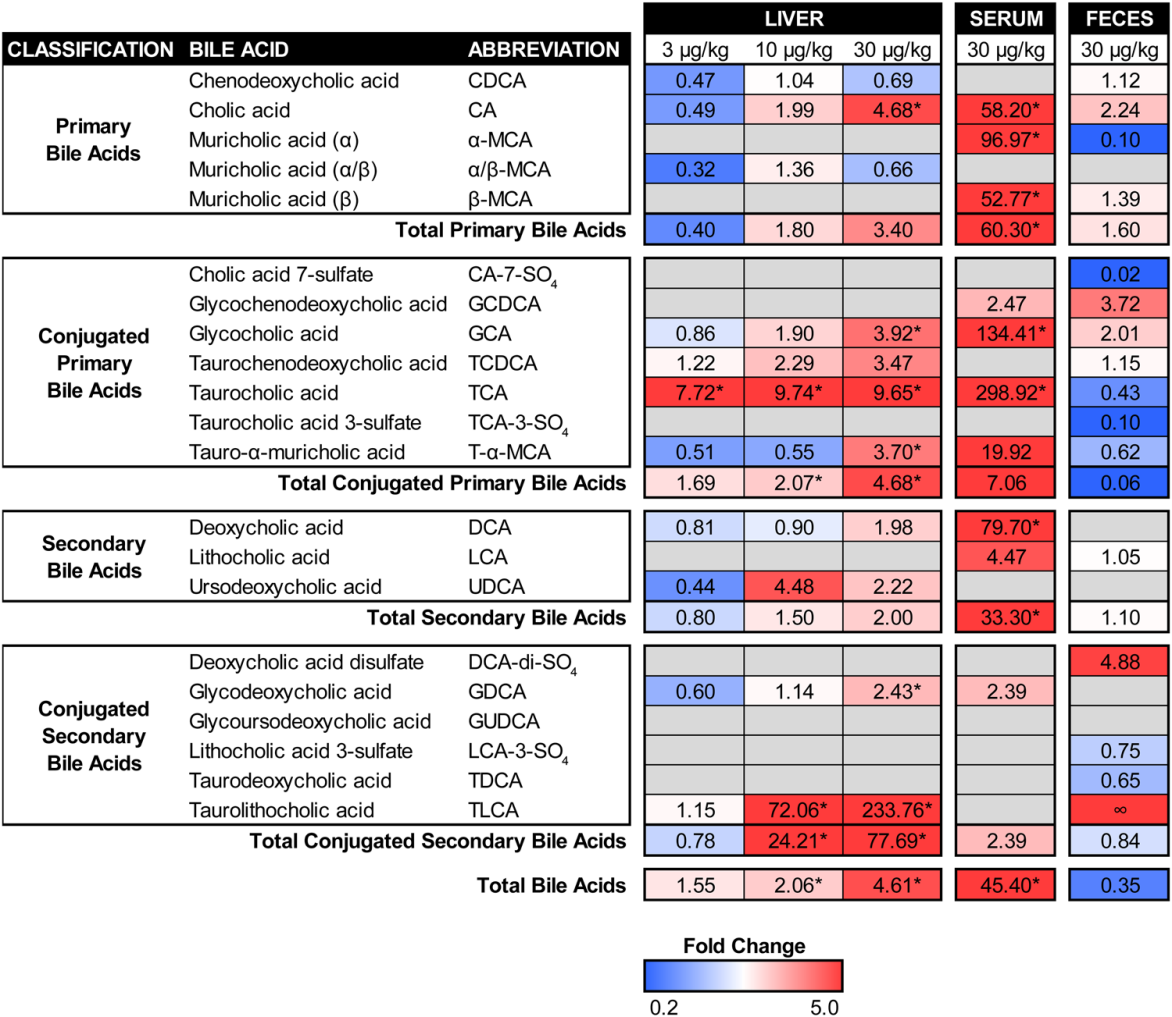
Bile acid profiles for the liver, serum, and feces of male C57BL/6 mice orally gavaged with sesame oil vehicle or TCDD (3–30 µg/kg) every 4 days for 28 days. Individual bile acid species were quantified using high resolution accurate mass liquid chromatography mass spectrometry and expressed as fold changes relative to vehicle controls. Red and blue indicate increased or decreased levels, respectively, while grey indicates ‘not detected’. ‘∞’ indicates an incalculable increase as the bile acid was detected in treated animals but not controls. Statistical significance (*p ≤ 0.05) was determined using one-way ANOVA analysis followed by Dunnett’s post-hoc test (liver) or a Student’s t-test (serum) performed in SAS 9.3. Fecal pellets were collected from two cages of co-housed mice for each treatment group (n = 2) and therefore statistical analysis was not performed.

HRAM-LC-MS analysis detected a dose-dependent increase in total hepatic bile acids, with 2.1- and 4.6-fold increases at 10 and 30 µg/kg TCDD, respectively (Fig. 4). CA, the predominant primary hepatic bile acid in control samples, increased 4.7-fold following treatment with 30 µg/kg TCDD. α/β-MCA was the second most abundant primary bile acid in controls, while hepatic CDCA levels were negligible. TCDD had no effect on hepatic α/β-MCA or CDCA, but dose-dependently increased total hepatic conjugated primary bile acid levels (max 4.7-fold), including glycocholic acid (GCA; 3.9-fold), taurocholic acid (TCA; 9.7-fold), and tauro-α-muricholic acid (T-α-MCA; 3.7-fold) (Fig. 4). Paradoxically, hepatic expression of *Baat*, which is responsible for hepatic taurine- and glycine-conjugation of bile acids, was repressed 12.0-fold (Fig. 3). In female mice, TCDD increased hepatic taurine (max 1.4-fold; *p* = 0.05) and glycine (max 20.3-fold; *p* = 0.10) levels^36^. TCDD repressed hepatic expression of taurine biosynthesis genes including cysteine dioxygenase 1 (*Cdo1*; 2.7-fold) and cysteine sulfinic acid decarboxylase (*Csad*; 4.5-fold), while the taurine transporter *Slc6a6* (encoding TAUT) was induced 1.8-fold (Supplementary Table S1), suggesting hepatic taurine uptake rather than *de novo* synthesis. Furthermore, TCDD elicited a dose-dependent increase in total hepatic levels of conjugated secondary bile acids (max 77.7-fold), suggesting increased bacterial metabolism. More specifically, TLCA, the most induced hepatic bile acid, was dose-dependently increased 72.1- and 233.8-fold at 10 and 30 µg/kg TCDD, respectively (Fig. 4).

Individual bile acids differ in their capacity to bind hepatic receptors. TLCA is the most potent endogenous activator of GPBAR1, a metabotropic bile acid receptor located on non-parenchymal cells including Kupffer cells, sinusoidal epithelial cells, and bile duct epithelial cells (cholangiocytes)^28^. Using qRT-PCR, hepatic *Gpbar1* expression was induced 4.3-fold at 30 µg/kg TCDD (Supplementary Fig. S2). Increased hepatic TLCA levels combined with *Gpbar1* induction would promote activation of GPBAR1 following TCDD treatment. Likewise, CDCA is the most potent endogenous FXR agonist, while T-α-MCA and T-β-MCA are competitive antagonists^37^. At low hepatic CDCA levels due to conversion to α-MCA, TCDD-elicited increases in T-α-MCA levels may inhibit FXR activation. Moreover, TCDD repressed hepatic *Nr1h4* (encodes FXR) 3.2-fold, further inhibiting the FXR signaling pathway (Supplementary Table S1).

Total bile acids in serum increased 45.4-fold at 30 µg/kg TCDD (Fig. 4). Taurine- and glycine-conjugates of CA exhibited increases of 298.9- and 134.4-fold, respectively. CA, α-MCA, and β-MCA were the most abundant species present in treated serum samples with increases of 58.2-, 97.0-, and 52.8-fold, respectively. Additionally, the secondary bile acid deoxycholic acid (DCA) was increased 79.7-fold.

Total fecal bile acids decreased 2.8-fold at 30 µg/kg TCDD (Fig. 4), suggesting decreased bile secretion from the gallbladder and/or increased intestinal reabsorption. Fecal levels of cholic acid 7-sulfate (CA-7-SO_4_) and taurocholic acid 3-sulfate (TCA-3-SO_4_) were decreased 55.2- and 10.3-fold, respectively, while deoxycholic acid disulfate (DCA-di-SO_4_) levels increased 4.9-fold. The former is consistent with a 267.5-fold repression of *Sult2a8*, the predominant sulfotransferase in the male liver based on RNA-Seq read counts (Supplementary Table S1). TCDD also decreased α-MCA fecal levels 10.3-fold, while TLCA levels changed from undetectable in controls to detectable in treated mice.

### Bile Acid Hydrophobicity

**Figure 5:**
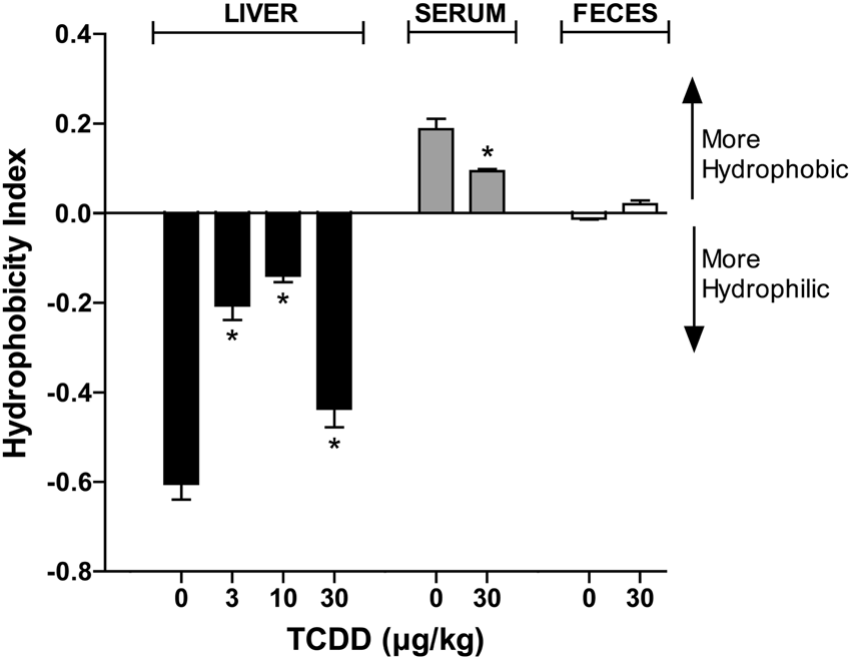
Hydrophobicity indices (HIx) of bile acid mixtures in the liver, serum, and feces of male C57BL/6 mice orally gavaged with sesame oil vehicle or TCDD (3–30 µg/kg TCDD) every 4 days for 28 days. Bile acid toxicity is positively associated with the degree of hydrophobicity, where increasingly positive and negative HIx values indicate greater hydrophobicity and hydrophilicity, respectively. Bars represent the average ± standard error of the mean for at least 4 biological replicates for liver and serum, or 2 pooled samples for feces. Statistical significance (*p ≤ 0.05) was determined using one-way ANOVA analysis followed by Dunnett’s post-hoc test (liver) or a Student’s t-test (serum) performed in SAS 9.3. Fecal pellets were collected from two cages of co-housed mice for each treatment group (n = 2) and therefore statistical analysis was not performed.

In general, bile acid toxicity increases with hydrophobicity^38, 39^, which depends on ionization, hydroxylation, and conjugation (taurine, glycine, sulfate, glucoronate)^40^. The potential toxicity of a bile acid mixture can be estimated by calculating the hydrophilic-hydrophobic balance using the hydrophobicity index (HIx) of each bile acid species in the mixture, as described by Heuman^41^. The HIx of TCA was arbitrarily set to 0, with increasingly positive and negative values assigned to species exhibiting relatively greater hydrophobicity and hydrophilicity, respectively. TCDD (3–30 µg/kg) shifted the hepatic bile acid mixture HIx toward greater hydrophobicity (Fig. 5), suggesting increased hepatotoxic potential. Each bile acid’s contribution to the overall HIx is dependent on its relative abundance within the mixture, and therefore low abundance species such as TLCA have lower impact. Consequently, the shift towards increased hydrophobicity is primarily driven by a decrease in the relative abundance of T-α-MCA, a highly hydrophilic species. In contrast, the serum bile acid mixture became more hydrophilic, while the fecal mixture exhibited a moderate increase in hydrophobicity (Fig. 5).

CYP3A enzymes play a role in bile acid metabolism by catalyzing 6α-, 7α-, and 6β-hydroxylation^42, 43^. TCDD repressed all hepatic *Cyp3a* isoforms including *3a13*, *3a25*, *3a16*, *3a11*, *3a59*, *3a44*, and *3a57* (17.0-, 6.0-, 5.2-, 4.9-, 4.4-, 3.0-, 2.4-fold), as well as ileal *Cyp3a44* (2.2-fold) (Supplementary Table S1). *Cyp3a* repression may decrease bile acid hydroxylation, thereby increasing the hydrophobicity of the bile acid pool.

### Enterohepatic Circulation

**Figure 6:**
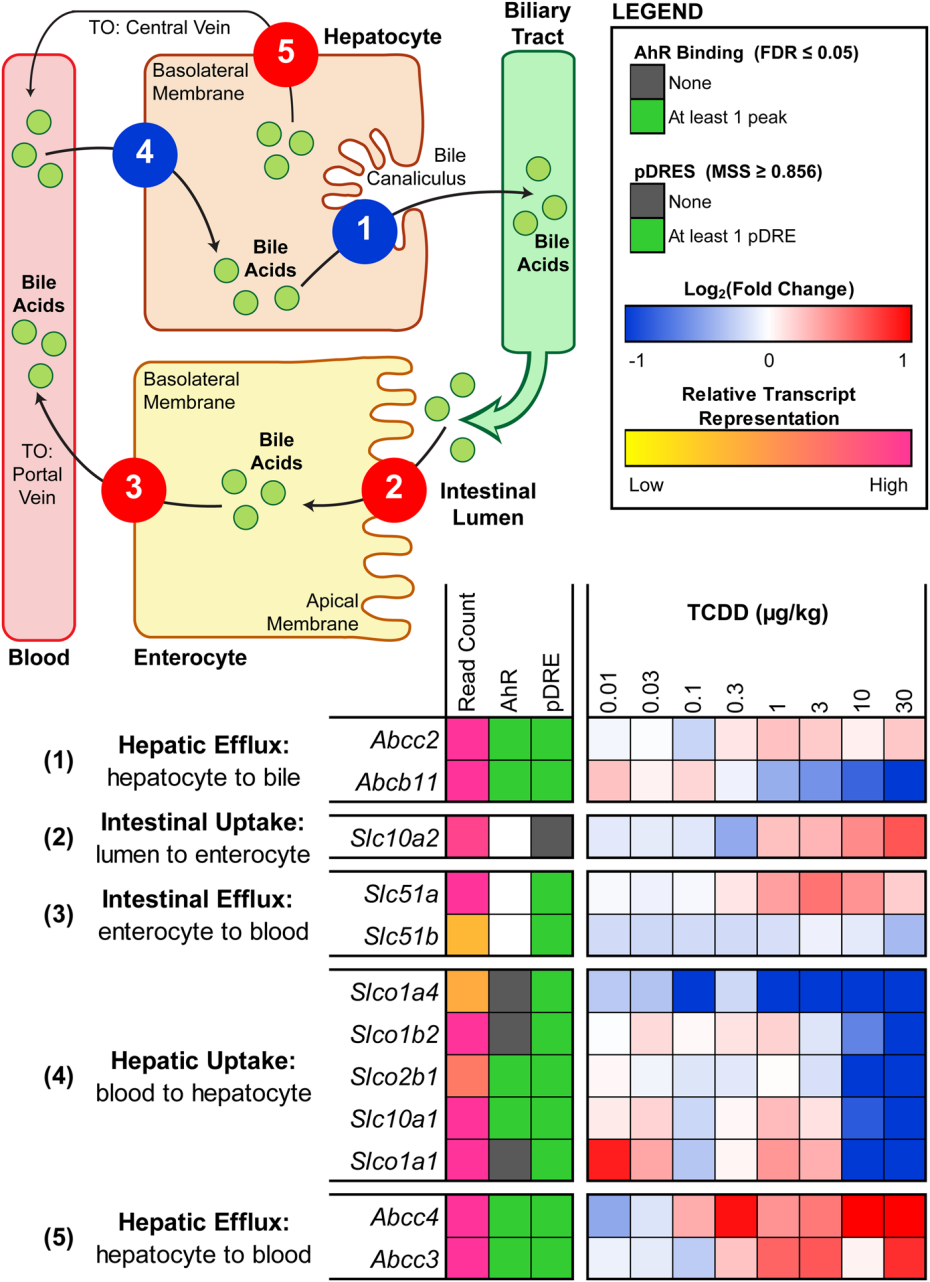
TCDD-elicited differential expression of bile acid transporters in the liver and ileum of male C57BL/6 mice orally gavaged with sesame oil vehicle or 0.01–30 µg/kg TCDD every 4 days for 28 days. Color scale represents the log_2_(fold change) for differential gene expression relative to vehicle controls, as determined through RNA-Seq analysis (n = 3). The numeric labels to the left of the heat map correspond to the numbered transport functions in the pictorial representation, where the color of each function represents the overall direction of differential expression exhibited by the group of transporters involved (i.e. induced = red vs. repressed = blue). The presence of pDREs (MSS ≥ 0.856) and hepatic AhR enrichment peaks (FDR ≤ 0.05) at 2 h are shown as green boxes. Read count represents the raw number of aligned reads to each transcript indicating potential level of expression, where yellow represents a lower level of expression (≤500 reads) and pink represents a higher level of expression (≥10,000).

Following synthesis, conjugated primary bile acids are secreted into the bile canaliculi by canalicular bile salt export pump (BSEP; aka ABCB11) and ABCC2. TCDD repressed hepatic *Abcb11* 4.7-fold, while *Abcc2* was unchanged (Fig. 6). Reduced hepatocyte-to-canaliculi export, combined with increased levels of hepatic conjugated primary bile acids likely caused spill-over into the sinusoidal blood and systemic circulation. Conjugated primary bile acids stored in the gallbladder are released into the duodenum in response to the intestinal hormone cholecystokinin following a meal, and progressively pass through the small intestine. Upon reaching the ileum, they are taken up into enterocytes by apical SLC10A2, and exported to the portal circulation by basal SLC51A and SLC51B. TCDD induced ileal expression of *Slc10a2* and *Slc51a* 1.6- and 1.5-fold, respectively, while ileal *Slc51b* was unchanged (Fig. 6). Induction of these intestinal transporters likely promotes active reabsorption of bile acids across the ileum. Circulating bile acids are then transported into hepatocytes by *Slc10a1*, *Slco1a1*, *Slco1a4*, *Slco1b2*, and *Slco2b1*, which were repressed 17.9-, 1186.3-, 2.5-, 7.9-, and 12.9-fold, respectively, by TCDD (Fig. 6). Specifically, SLC10A1 is primarily responsible for taurine- and glycine-conjugated bile acid uptake, while SLCO1B2 plays a key role in unconjugated bile acid uptake^44, 45^. *Abcc3* and *Abcc4*, which are responsible for bile acid efflux from hepatocytes into systemic circulation, were induced 1.8- and 58.1-fold at 30 µg/kg TCDD (Fig. 6). Collectively, repression of hepatic importers combined with induction of hepatic exporters likely contributed to the 45.4-fold increase in total serum bile acids.

**Figure 7:**
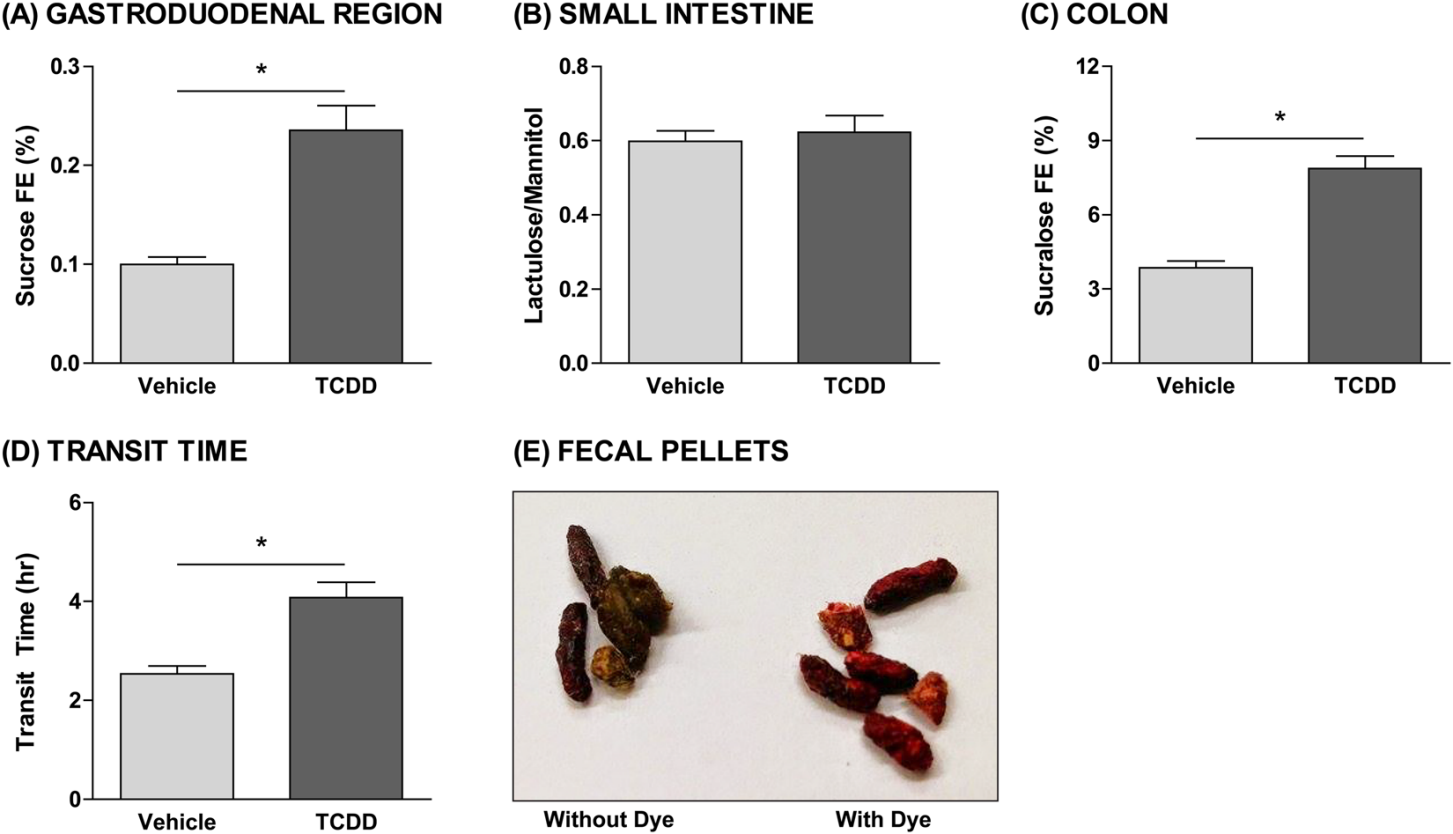
Effects of TCDD on intestinal permeability and motility. Para-cellular permeability of the (**A**) gastroduodenal region, (**B**) small intestine, and (**C**) colon in male C57BL/6 mice orally gavaged with sesame oil vehicle or 30 µg/kg TCDD every 4 days. At 26 days after the initial dose, mice were orally gavaged with sucrose, lactulose, mannitol, and sucralose to assess segment-specific intestinal permeability. Urine was collected over a 5-hour period, and fractional excretion (FE) of the probes was measured using ultra high performance liquid chromatography (UPLC) tandem mass spectrometry (MS/MS). (**D**) Whole gut transit time of male C57BL/6 mice orally gavaged with sesame oil vehicle or 30 µg/kg TCDD every 4 days. At 27 days after the initial dose, transit time was monitored by orally gavaging mice with Carmine Red and then measuring the interval between the gavage and the first observance of dye in the fecal pellets. (**E**) Fecal pellets collected from mice following oral gavage with Carmine Red dye (right) compared to pellets lacking the dye (left). Bars represent the average ± standard error of the mean for at least 6 biological replicates. Statistical significance (*p ≤ 0.05) was determined using a Student’s t-test performed in SAS 9.3.

Approximately 95% of bile acids secreted into the intestinal lumen are reabsorbed with the remaining 5% lost via fecal excretion^18^. Highly efficient enterohepatic circulation allows the liver to maintain a low rate of *de novo* primary bile acid biosynthesis. At 30 µg/kg TCDD, sugar probe analysis revealed a 2.4- and 2.0-fold increase in gastroduodenal and colonic para-cellular (i.e. between enterocytes) permeability, respectively, while the permeability of the small intestine was unaffected (Fig. 7A–C). Furthermore, whole gut transit time increased from 2.5 h in controls to 4.1 h in 30 µg/kg TCDD-treated mice, indicating a 1.6-fold reduction in gut motility (Fig. 7D,E). Induction of active ileal transporters (*Slc10a2*, *Slc51a*), increased passive para-cellular duodenal and colonic transport, and decreased gut motility are in accordance with increased intestinal reabsorption and decreased fecal excretion of bile acids. These changes in intestinal function are also consistent with TCDD-elicited increases in total serum bile acids and decreases in total fecal bile acids.

### Microbial Bile Acid Metabolism

Bile acids not reabsorbed by ileal enterocytes are metabolized by microbiota in the colon, the intestinal segment with the densest and most diverse microbial population. Various bacterial genera metabolize primary and conjugated primary bile acids to yield “secondary” bile acids. Bacterial bile salt hydrolase (BSH) cleaves the C24 N-acyl amide bond of conjugated bile acids, removing the glycine or taurine moiety. Several gut bacterial genera express *bsh* including *Bacteroides*, *Bifidobacterium*, *Clostridium*, *Lactobacillus*, and *Listeria* ^46^. The exposed 7-hydroxyl groups of unconjugated CA and CDCA are susceptible to bacterially-catalyzed 7α-dehydroxylation, yielding the secondary bile acids DCA and lithocholic acid (LCA), respectively. Bile acid 7α-dehydroxylation is a multi-step enzymatic process involving a set of genes encoded within the bile acid-inducible (*bai*) operon including *baiCD*, the oxidoreductase enzyme which catalyzes 3-dehydro-4-bile acid oxidation^47^. To date, 7α-dehydroxylation activity has only been identified in the *Clostridium* and *Eubacterium* genera^46^.

**Figure 8:**
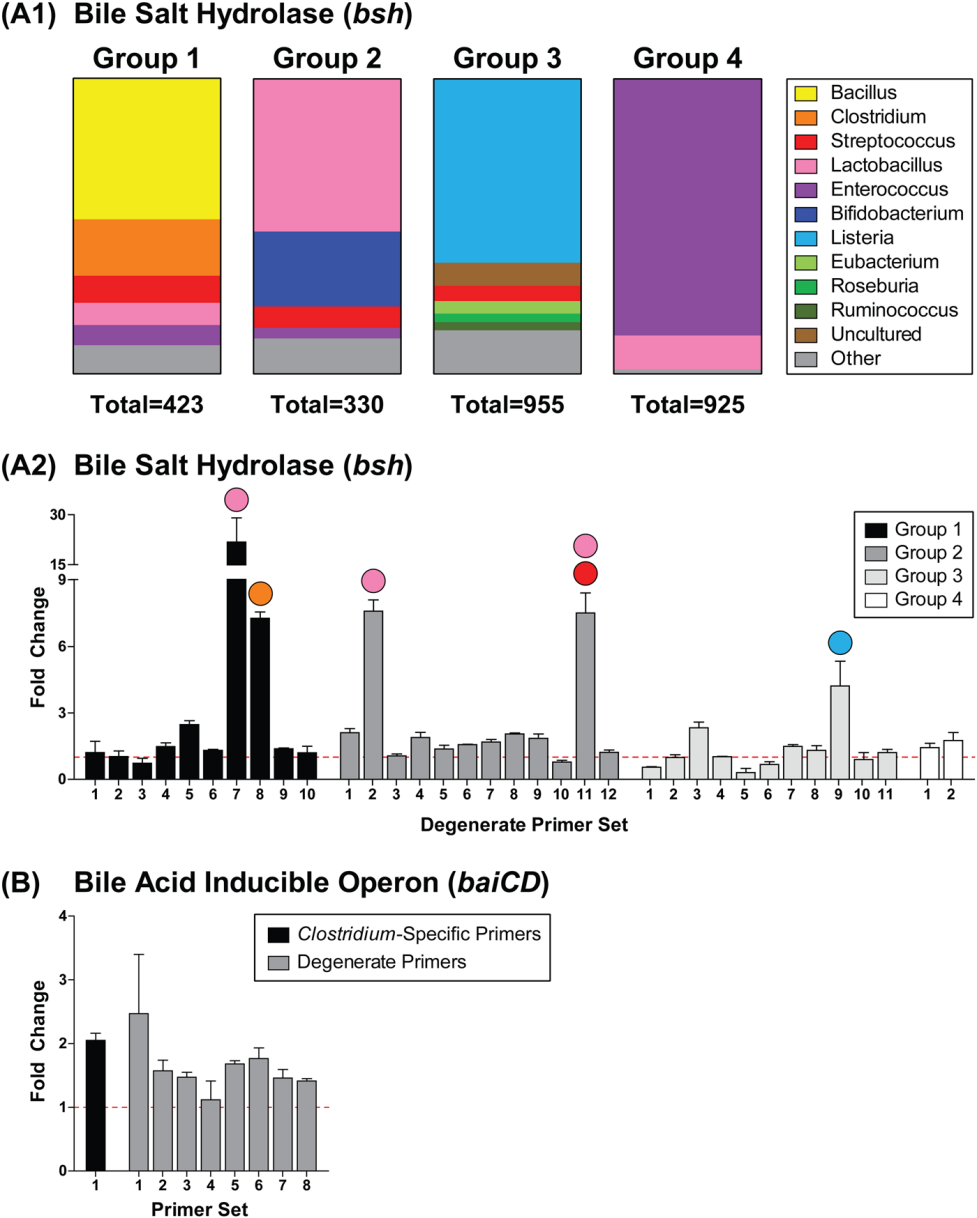
Effects of TCDD on bacterial genes involved in bile acid metabolism. (**A1**) Bacterial classifications (genus level) of the *bile salt hydrolase* (*bsh*) sequences in each phylogenetic group, which were separated based on nucleotide similarity. Degenerate qRT-PCR analysis of (**A2**) *bsh* and (**B**) bile acid-inducible operon (*baiCD*) gene levels in the fecal pellets of male C57BL/6 mice orally gavaged with sesame oil vehicle or 30 µg/kg TCDD every 4 days for 28 days. Fecal levels of *baiCD* were also quantified using a primer set previously validated to target several *Clostridium* species which exhibit 7α-dehydroxylation activity (B: black bar). Colored circles above highly increased *bsh* primer sets indicate the bacterial genus which is primarily targeted. Values were normalized to levels of the 16S ribosomal RNA gene. Bars represent the average fold change relative to vehicle controls ± standard error of the mean. Fecal pellets were collected from two cages of co-housed mice for each treatment group (n = 2) and therefore statistical analysis was not performed.

**Figure 9:**
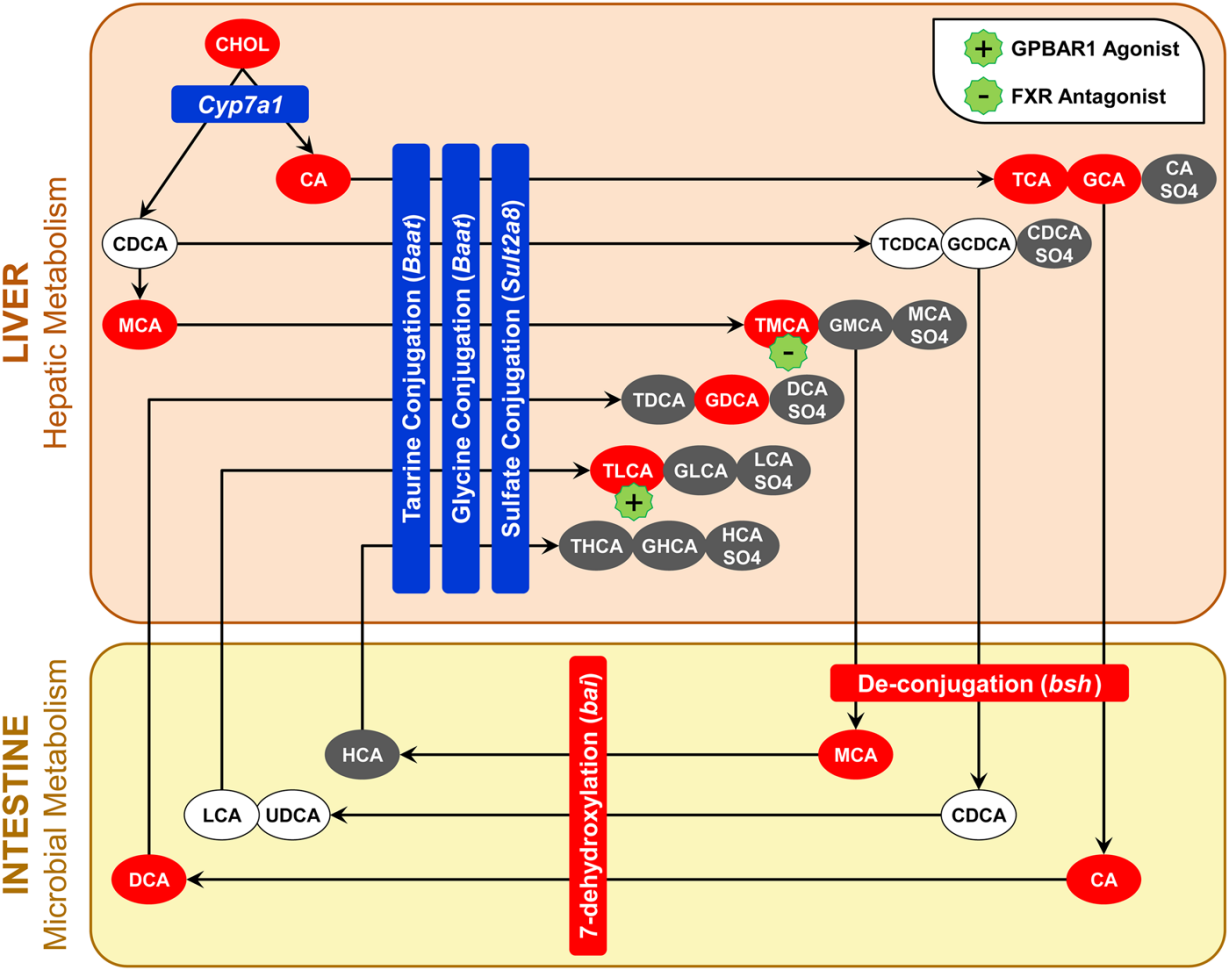
The effect of TCDD on the hepatic and microbial metabolism of individual bile acid species in male C57BL/6 mice orally gavaged with sesame oil vehicle or TCDD every 4 days for 28 days. Ovals represent bile acid species, while rectangles represent genes involved in bile acid metabolism. Placement of genes within the ‘liver’ and ‘intestine’ boxes represents the location of the metabolic reaction and the source of the enzyme involved. Red represents ‘increased levels’, blue represents ‘decreased levels’, white represents ‘no change’, and grey represents ‘not detected’. The color of each bile acid depicts the direction of change within the liver and/or serum relative to vehicle controls.

As opposed to evaluating changes in the abundance of specific bacterial species associated with bile acid metabolism, we examined changes in the levels of *bsh* and *baiCD*, the loci responsible for bile acid deconjugation and dehydroxylation, respectively. Bacterial DNA extracted from fecal pellets was analyzed by qRT-PCR using computationally-designed degenerate primer sets. Overall, *bsh* gene levels within phylogenetic groups 1, 2, and 3 were increased, while levels within phylogenetic group 4 were unaffected by TCDD (Fig. 8A2). In particular, primer sets 7 and 8 within group 1 increased 21.8- and 7.3-fold, respectively, while primer sets 2 and 11 within group 2 increased 7.6- and 7.5-fold, respectively, and primer set 9 within group 3 increased 4.2-fold at 30 µg/kg TCDD. These primer sets primarily target *Lactobacillus* (Group1-Primer7; Group2-Primer2; Group2-Primer11), *Clostridium* (Group1-Primer8), *Streptococcus* (Group2-Primer11), and *Listeria* (Group3-Primer9) species, suggesting TCDD increased the relative abundance of several species within these genera. Primers targeting *Clostridium baiCD* ^48, 49^ identified a 2.1-fold increase at 30 µg/kg TCDD (Fig. 8B), suggesting increased relative abundance of *Clostridium* strains known to exhibit 7α-dehydroxylation activity. Similarly, 7 of the 8 degenerate primer sets designed to target *baiCD* across the gut microbiome exhibited increased levels (1.5- to 2.5-fold). Consistent with these results, TCDD is reported to increase the abundance of several *Lactobacillus* and *Clostridium* species in the mouse gut microbiota^50^. Together, these results suggest that TCDD increased deconjugation and 7α-dehydroxylation activity within the gut microbiota, consistent with increased secondary bile acids such as DCA in the serum and increased conjugated secondary bile acids such as TLCA and GDCA in the liver (Fig. 9).

## Discussion

The effects of TCDD and related compounds on bile acid levels have been previously reported in a wide variety of rodent models using various treatment regimens^22, 23, 26, 30, 51, 52^. This study further elucidates AhR-mediated dysregulation of bile acid homeostasis in C57BL/6 mice by mapping complementary metabolomics, RNA-Seq, histopathology, and phenotypic data to bile acid biosynthesis, transport, and metabolism pathways. We demonstrate that TCDD altered primary and secondary bile acid profiles, consistent with changes in bile acid transport, gut microbiota metabolism, and intestinal permeability and motility.

In this study, TCDD dose-dependently increased total bile acid levels in the liver and serum, as previously reported in rodent studies involving AhR agonists^22, 23, 30, 51^. This is in accordance with the hepatic accumulation of cholesterol and cholesterol-esters, and may represent an adverse consequence of the liver’s attempt to minimize potential lipotoxicity through the removal of excess cholesterol. Paradoxically, the majority of genes associated with bile acid biosynthesis exhibited dose-dependent repression including the rate-limiting enzyme *Cyp7a1*, *Cyp7b1* of the alternative pathway, and *Baat*, which catalyzes taurine/glycine-conjugation. AhR-mediated repression of *Cyp7a1* has been previously reported^13, 26, 27, 52^, although TCDF is reported to induce *Cyp7a1* mRNA and increase CYP7A1 protein^51^. Interestingly, AhR binding was enriched upstream of the *Cyp7a1* transcription start site in both male and female mice, in the absence of a pDRE.

Bile acid biosynthesis is regulated by negative feedback involving FXR activation. Bile acid-activated FXR induces hepatic *Nr0b2* (encodes SHP), which interacts with LRH-1 and inhibits *Cyp7a1* expression^53^. Moreover, in the distal ileum, activated FXR induces *Fgf15* which binds to hepatic FGFR4/KLB to inhibit *Cyp7a1* expression. However, *Nr1h4* (encodes FXR), *Nr0b2*, *Fgfr4*, and *Klb* were all repressed by TCDD, while *Fgf15* exhibited no clear expression pattern. Related AhR agonists including TCDF and flutamide also repress *Nr1h4* and *Nr0b2* ^51, 54^. Furthermore, hepatic CDCA levels, the most potent FXR agonist, were negligible, while TCDD increased hepatic levels of T-α-MCA, a competitive FXR antagonist. Together, these results suggest that TCDD disrupts bile acid-induced feedback inhibition possibly by inhibiting the FXR signaling pathway.

In contrast to biosynthesis, TCDD-elicited differential expression of genes associated with enterohepatic circulation and transport is consistent with serum and hepatic bile acid accumulation. As previously reported^27, 55^, TCDD repressed hepatic *Abcb11* (*Bsep*), which is responsible for transporting newly synthesized primary bile acids from hepatocytes to bile canaliculi. This would impair bile collection within the gallbladder, causing accumulation within hepatocytes and eventual spill over into sinusoidal blood. Inhibition of FXR signaling may be responsible for repression of *Abcb11*, a known FXR target gene. TCDD also repressed transporters involved in hepatic uptake from the blood (*Slco1a1*, *Slc10a1*, *Slco2b1*, *Slco1b2*, and *Slco1a4*) and induced hepatic efflux transporters (*Abcc4*, *Abcc3*). Accordingly, bile acid accumulation was greater in the serum compared to the liver. Serum bile acid accumulation may also be facilitated by enhanced enterocyte uptake (*Slc10a2*) and efflux (*Slc51a*), as well as passive reabsorption due to reduced gut motility and increased intestinal para-cellular permeability. As a result, TCDD reduced fecal bile acid levels, although impaired gallbladder emptying may also be a contributing factor. Overall, AhR-mediated differential expression of bile acid transporters is consistent with bile acid accumulation, as well as reduced bile flow and biliary excretion in TCDD-treated rodents^24^.

Increased secondary bile acid levels provide indirect evidence of microbial bile acid metabolism and changes in the gut microbiota. qRT-PCR analysis of bacterial bile acid metabolism loci revealed that TCDD increased fecal levels of *bsh* and the *bai* operon, suggesting increased gut microbiota deconjugation and dehydroxylation activity. Accordingly, serum levels of secondary bile acids such as DCA and hepatic levels of conjugated secondary bile acids including TLCA and GDCA were increased by TCDD. Decreased gut motility may also facilitate bacterial metabolism and the passive reabsorption of hydrophobic secondary bile acids.

TCDD shifted the hepatic HIx towards increased hydrophobicity, which correlates with hepatotoxicity due to membrane disruption, ROS generation, induction of pro-inflammatory mediators, and the activation of necrotic signaling. Specifically, TLCA, a conjugated secondary bile acid species with a hydrophobicity index of 1.00, exhibited the greatest hepatic increase in response to TCDD. TLCA induces cholestasis by impairing hepatobiliary exocytosis, inhibiting the insertion of transport proteins into apical membranes, and disrupting bile flow^29, 56, 57^. It is also the most potent endogenous agonist of GPBAR1, a metabotropic receptor highly expressed on bile duct cholangiocytes^28^. TLCA activation of GPBAR1 induces cholangiocyte proliferation through: (i) generation of ROS which activate Rous sarcoma oncogene (cSrc), epidermal growth factor receptor (EGFR), and ERK1/2, and (ii) inhibition of apoptosis through phosphorylation of the CD95 death receptor^29^. Therefore, increased levels of hepatic TLCA and other hydrophobic bile acids may contribute to TCDD-elicited hepatotoxicity by inducing bile duct proliferation and impairing bile flow.

Collectively, these results suggest that bile acid accumulation, enhanced enterohepatic circulation, and alterations in microbial metabolism contribute to TCDD-elicited hepatotoxicity and the progression of hepatic steatosis to steatohepatitis with fibrosis. Numerous studies involving whole-body AhR knockout mice have demonstrated that AhR activation is required for TCDD-elicited hepatotoxicity including bile duct proliferation^15, 58^. Other AhR ligands (TCDF, OCDD, PeCDF, and flutamide) also dysregulate bile acid homeostasis^22, 23, 51, 54^. The presence of DREs, AhR enrichment, and dose-dependent induction/repression of key genes associated with bile acid homeostasis, provide compelling evidence that these effects are AhR mediated. However, hepatocyte-specific conditional AhR knockout studies would be required to unequivocally demonstrate the role of AhR activation in TCDD-elicited dysregulation of bile acid homeostasis.

In addition to effects on bile acid homeostasis, TCDD also disrupts other hepatic processes and metabolic pathways including lipid metabolism^13, 59^, iron homeostasis and heme metabolism^33^, extracellular matrix deposition and remodeling^16^, and antioxidant defenses^36^. Accumulating evidence suggests AhR-mediated hepatotoxicity is a cumulative response to the overall burden of multiple disrupted metabolic pathways, rather than a single event. Further studies are required to determine whether TCDD and related compounds elicit comparable metabolic effects in human models.

## Materials and Methods

### Animal Handling and Treatment

Postnatal day 25 (PND25) male C57BL/6 mice weighing within 10% of each other were obtained from Charles River Laboratories (Kingston, NY) and housed in Innovive Innocages (San Diego, CA) containing ALPHA-dri bedding (Shepherd Specialty Papers, Chicago, IL) in a 23 °C environment with 30–40% humidity and a 12-hour light/dark cycle (7am–7 pm). Mice were provided Aquavive water (Innovive) and Harlan Teklad 22/5 Rodent Diet 8940 (Madison, WI) *ad libitum*, and were acclimated for 4d prior to treatment. Beginning on PND28, animals (n = 8; co-housed 4 per cage) were orally gavaged with sesame oil vehicle (Sigma-Aldrich, St. Louis, MO), 0.01, 0.03, 0.1, 0.3, 1, 3, 10, or 30 μg/kg TCDD (AccuStandard, New Haven, CT) every 4d for a total of 28d (7 exposures; Supplementary Fig. S3). The doses used compensate for the relatively short study duration compared to lifelong cumulative human exposure from diverse AhR ligands, the bioaccumulative nature of halogenated AhR ligands, and differences in TCDD’s metabolism and half-life (humans: 1–11 years^60, 61^, mice: 8–12d^62^). TCDD doses between 0.01 and 30 µg/kg result in mouse hepatic tissue levels that span human background serum concentrations reported in the United States, Germany, Spain, and the United Kingdom as well as serum levels in Viktor Yushchenko 4–39 months following intentional poisoning^16^. For the histopathological comparison, female C57BL/6 mice were orally gavaged with sesame oil vehicle or 0.01–30 µg/kg TCDD every 4d for 28d, as previously described^32^. All animal handling procedures were performed with the approval of the Michigan State University (MSU) Institutional Animal Care and Use Committee, in accordance with the relevant ethical guidelines and regulations.

### Sample Collection

At 25d after the initial dose (PND53), fecal pellets from the previous 24 hours (h) were collected from each cage (4 mice per cage) and stored at −80 °C. At 28d after the initial exposure (PND56), mice (fasted for 6 h) were weighed and blood was collected from the submandibular vein prior to cervical dislocation. The gallbladder length, width, and depth were measured using a micro-caliper. Its volume was calculated assuming an ellipsoid shape: volume (µL) = length (mm) × width (mm) × depth (mm) × π/6^63, 64^. The distal ileum (10 cm section extending from the caecum toward the jejunum) was removed, flushed with Ca^2+^/Mg^2+^-free phosphate buffered saline (PBS; Sigma), and opened longitudinally. The epithelial lining was scraped into a vial containing TRIzol (Invitrogen, Carlsbad, CA), frozen in liquid nitrogen, and stored at −80 °C. Liver, gonadal white adipose tissue (gWAT), and brown adipose tissue were removed, weighed, frozen in liquid nitrogen, and stored at −80 °C. Serum alkaline phosphatase (ALP) activity was determined using a commercial kit (Pointe Scientific, Canton, MI).

### Histopathology

All processing was performed by the MSU Investigative Histopathology Laboratory (https://humanpathology.natsci.msu.edu/). Formalin-fixed hepatic tissues were vacuum infiltrated with paraffin using a Tissue-Tek VIP 2000 tissue processor (Sakura) and embedded with the Thermo Fisher HistoCentre III Embedding Center (Thermo Fisher, Waltham, Massachusetts). Paraffin blocks were sectioned at 4–5 µm with a Reichert Jung 2030 rotary microtome (Reichert, Depew, New York) and dried for 2–24 h at 56 °C to ensure adherence to the slides. For lipid staining, liver sections frozen in O.C.T. compound were sectioned at 6 µm, fixed in 10% NBF for 5 min, rinsed with water, and immersed in 100% propylene glycol for 5 min. Liver sections were stained with hematoxylin and eosin (H&E) for general histological assessment. Lipids were stained using Oil Red O (ORO) as previously described^65^. Macrophages were labeled using a monoclonal anti-mouse F4/80 antibody (1:100 dilution; AbD Serotec, Raleigh, NC). Collagen was stained with 0.1% Picro-Sirius Red (PSR) dye, with Weigert’s Hematoxylin nuclear dye. Histological assessment of hepatic tissue from female C57BL/6 mice was performed as previously described^16, 32^.

Quantitation of ORO staining was performed using the Quantitative Histological Analysis Tool (QuHAnT)^66^. Briefly, slides were digitized at 20x magnification using an Olympus Virtual Slide System VS110 (Olympus, PA) and images were sampled at 100% coverage using the Visiomorph Microimager (Visiopharm, Denmark). Using ImageJ (http://rsb.info.nih.gov.ij/), optimal hue, saturation, and value (HSV) image segmentation thresholds for feature extraction were determined to be 0 to 50 and 225 to 255 (hue), 125 to 255 (saturation), and 0 to 255 (value), while optimal total tissue feature extraction thresholds were 0 to 255 (hue), 20 to 255 (saturation), and 0 to 255 (value). Volume densities were estimated as the sum of positive hits (P_positive_ staining) divided by the total number of tissue hits (P_tissue_) for each section: V_v_ = (P_positive_ staining/P_tissue_) × 100.

### RNA-Seq

Frozen ileal epithelium scrapings and liver samples were homogenized in TRIzol (Ambion, Waltham, MA) using a Mixer Mill 300 tissue homogenizer (Retsch, Germany) and total RNA was isolated as previously described^13^. RNA was quantified using a Nano-drop spectrophotometer (Thermo Scientific, Wilmington, DE) at 260 nm, and purity was assessed using the A_260_/A_280_ ratio and the Caliper LabChip GX (Perkin Elmer, Waltham, MA).

Dose-dependent gene expression was examined using RNA-Seq performed at the MSU Research Technology Support Facility (RTSF) Genomics Core (rtsf.natsci.msu.edu/genomics). Ileal libraries from three independent biological replicates (n = 3) were prepared using the Ovation Mouse RNA-Seq System 1–16 sample preparation kit (NuGen, San Carlos, CA), with an additional DNase step. Libraries were quantified and sequenced as previously described, using a read depth of ~30 M per sample^33, 67^. Quality was determined using FASTQC v0.11.3 and adaptor sequences were removed using Trimmomatic v0.33. Reads were mapped to the mouse reference genome (GRCm38 release 81) using Bowtie2 v2.2.6 and TopHat2 v2.1.0. Alignments were converted to SAM format using SAMTools v1.2.0. For TCDD-mediated differential gene expression, fold changes were calculated relative to vehicle controls. Hepatic RNA-Seq analysis was previously published^33^. Genes were considered differentially expressed if |fold change| ≥ 1.5 and statistical P1(*t*) value ≥ 0.8 at one or more doses. RNA-Seq datasets for the ileal epithelium and liver were deposited in the Gene Expression Omnibus (GEO; accession number GSE89430 and GSE87519, respectively). The RNA-Seq results (fold changes, P1(*t*) values) for each gene discussed in the manuscript have been compiled in Supplementary Table S1. Dose-response modeling was performed using the ToxResponse Modeler^68^, and median effective dose (ED_50_) values for differentially expressed genes exhibiting a sigmoidal response were reported in Supplementary Table S1. Estimation of benchmark dose (BMD) and BMD lower confidence limit (BMDL) was performed using BMDExpress^69^ as previously described^32^, and has been included in Supplementary Table S1.

### Putative DRE Identification and Hepatic AhR ChIP-Seq

Putative dioxin response elements (pDREs) with matrix similarity scores (MSS) ≥ 0.856 were previously identified^36^. ChIP-Seq was previously performed on liver samples from male C57BL/6 mice 2 h after a single bolus oral gavage of 30 µg/kg TCDD^33, 70^. Briefly, cross-linked DNA was immunoprecipitated with either rabbit IgG or anti-AhR (H-211, sc-5579; Santa Cruz, CA). Libraries were prepared using the MicroPlex kit (Diagenode), pooled, and sequenced at a depth of a ~30 M on an Illumina HiSeq 2500 at the MSU RTSF Genomics Core. Reads were mapped to the mouse reference genome (GRCm38 release 81) using Bowtie 2.0.0 and alignments converted to SAM format using SAMTools v0.1.2. Normalization and peak calling was performed using CisGenome^71^, by comparing IgG control and AhR enriched samples (n = 5) using a bin size (−b) of 25 and boundary refinement resolution (−bw) of 1 with default parameters.

### Hepatic Quantitative Real-Time Polymerase Chain Reaction (qRT-PCR)

Given the low (≤10) read counts for *Gpbar1* in the RNA-Seq dataset, differential expression was confirmed by qRT-PCR^13^. Total hepatic RNA was reverse transcribed by SuperScript II (Invitrogen) using oligo dT primer according to the manufacturer’s protocol. PCR amplification was conducted on a Bio-Rad CFX Connect Real-Time PCR Detection System. Gene expression relative to vehicle control was calculated using the 2^−∆∆CT^ method, where each sample was normalized to the geometric mean of 3 housekeeping genes (*Actb*, *Gapdh*, and *Hprt*). Gene expression data are plotted relative to vehicle control. Forward and reverse primer sequences are provided in Supplementary Table S2.

### LC-MS Analysis of Bile Acids

Serum (10 µL) was added to 10 µL of a 20 ng/µL stock solution of deuterated (d4) glycochenodeoxycholic acid (GCDCA, from Steraloids, Inc.) in methanol, and extracted with ice-cold methanol as previously described^72^. Frozen liver samples (50 mg) or dried fecal pellets (50 mg) were combined with 10 µL of 20 ng/µL d4-GCDCA stock solution and 1.0 mL of ice-cold methanol^72^, and homogenized with 0.5 mm zirconium oxide beads using an air-cooled Bullet Blender (NextAdvance). Extracts were dried under vacuum, reconstituted in 2.0 mL of 50% methanol, filtered through 0.2 µm nylon membranes (Pall Life Sciences), and stored at −80 °C.

Bile acids were analyzed by high resolution/accurate mass (HRAM)-LC-MS using an Agilent 1260 capillary HPLC coupled to a Thermo Scientific LTQ-Orbitrap Velos mass spectrometer. Extracts (2 µL) were injected into water containing 0.1% formic acid, and loaded onto an inline peptide Opti-Trap (Optimize Technologies) for 3.0 min at 5 µL/min for desalting and concentration. Bile acids were then eluted by a 30 min water/acetonitrile gradient from 98% water containing 0.1% formic acid to 95% acetonitrile containing 0.1% formic acid (modified from ref. 73) and separated by a ProntoSil C18AQ 200 µm × 50 mm, 3 u column (nanoLCMS Solutions) at a flow rate of 2 µL/min. The column eluent was introduced to the LTQ-Orbitrap Velos mass spectrometer by an Advance nano-ESI source (Michrom BioResources) at a spray voltage of −1.7 kV. High resolution (R = 100,000 at 400 *m/z*) negative ion mass spectra were collected over 300–900 *m/z*. The ion transfer tube of the mass spectrometer was maintained at 250 °C, and the S-lens was set to 50%. Appropriate sample dilution factors were determined empirically to ensure a linear range of detector response^74^. Chromatographic peak alignment, feature detection, quantitation and bile acid identification were performed using MAVEN^75^ and SECD-LIMSA^76^ software. All bile acids were identified by comparison of retention time and accurate mass data against reference standards. Relative quantitation was performed against the deuterated GCDCA internal standard. TCDD-elicited changes in bile acid species are reported as fold changes relative to vehicle controls.

### Intestinal Function

At 26d after the initial dose (PND54), mice were fasted for 4 h (without access to food or water) prior to oral gavage with 150 µL of a solution containing 100 mg sucrose, 12 mg lactulose, 8 mg mannitol, and 6 mg sucralose. Urine from each mouse was collected over a 5 h period, flash frozen, and combined prior to analysis. Urine was diluted in 95% acetonitrile/5% water: 100-fold for mannitol detection, 200-fold for sucrose and lactulose detection, and 500-fold for sucralose detection. Sugar probe recovery was measured using ultra high performance liquid chromatography-tandem mass spectrometry (UPLC-MS/MS), performed at the MSU RTSF Mass Spectrometry and Metabolomics Core (https://rtsf.natsci.msu.edu/mass-spectrometry/). Diluted urine samples were injected onto an Acquity UPLC BEH Amide column (1.7 µm, 2.1 × 100 mm) (Waters, Milford, MA) maintained at 50 °C on a Waters ACQUITY UPLC systems (Waters). Mobile phase A was 50 mM ammonium acetate buffer (pH = 9.6) and mobile phase B was acetonitrile. Chromatographic separation was performed over 8 minutes (min) using a flow rate of 0.2 mL/min in the following gradient: time (t) = 0 min, 95% B; t = 1 min, 95% B; t = 2 min, 70% B; t = 5 min, 30% B; t = 5.01 min, 10% B; t = 6 min, 10% B; t = 6.01 min, 95% B; t = 8 min, 95% B. The autosampler was cooled to 10 °C, with an injection volume of 5 µL. UPLC was coupled with negative-mode electrospray ionization to a Waters Quattro Premier XE Mass Spectrometer (Waters) operating in multiple reaction monitoring (MRM) mode. The capillary voltage was −2.5 kV, while desolvation gas flow rate was 600 L/h. Source temperature and desolvation temperature were 120 °C and 450 °C, respectively. MRM parameters including cone voltage, collision voltage, precursor ion, and product ion were optimized by flow injection of pure standard for each individual compound (Supplementary Table S3).

Data analysis including peak integration was performed using MAVEN^75^. Waters raw data were converted to mzxml format using msconvert in the ProteoWizard Tools^77^. Fractional excretion of each probe was calculated using: (probe concentration from MS X total urine volume excreted)/probe input. Fractional sucrose excretion (%) was used to assess gastroduodenal permeability, the lactulose-to-mannitol ratio was used to assess small intestinal permeability, and fractional sucralose excretion (%) was used to assess colonic permeability^78, 79^.

At 27d after the initial dose (PND55), whole gut transit time (WGTT) was determined using carmine red dye, which cannot be intestinally absorbed. At time 0 (t_0_), mice were orally gavaged with 150 µL of carmine red solution (6% carmine red dye suspended in 0.5% methylcellulose). Fecal pellets were monitored continuously for the presence of carmine red. WGTT represents the time interval between t_0_ and the first observance of carmine red dye in the fecal pellets.

### Degenerate qRT-PCR of Bacterial Functional Genes

The *bsh* Hidden Markov Model (HMM) constructed by the Ribosomal Database Project consisted of 12 vetted seed sequences from *Lactobacillus*, *Clostridium*, *Roseburia*, *Eubacterium*, *Butyrivibrio*, and *Streptococcus* species, while the *baiCD* HMM consisted of 10 vetted seed sequences from *Clostridium* and *Lachnospiraceae* species. Using these models, more inclusive sequence sets were created for *bsh* and *baiCD* within the ‘FunGene functional gene pipeline & repository’^80^ by extracting sequences from GenBank. Setting the HMM consensus percent identity cut-off to ≥40%, nucleotide sequences of *bsh* and *baiCD* were obtained from 2633 and 129 bacterial organisms, respectively. Given the diversity of bacterial organisms found to express *bsh*, the 2633 sequences from FunGene were separated into 4 groups based on nucleotide similarity determined through construction of a phylogenetic tree (Group 1: 423, Group 2: 330, Group 3: 955, Group 4: 925). Bacterial classifications (genus level) of the *bsh* sequences in each phylogenetic group are depicted in Fig. 8A1. In contrast, the 129 *baiCD* sequences exhibited sufficient similarity and were therefore kept as a single composite group. The Primer Design terminal program developed by the Ribosomal Database Project (https://github.com/rdpstaff/PrimerDesign) was used to design degenerate primer sets which target conserved regions within *baiCD* and each phylogenetic group of *bsh* (Supplementary Table S2). To estimate the overall abundance of bile acid metabolism genes within the gut microbiome, primer sets were selected to target ≥75% of the *baiCD* sequences and the sequences within each *bsh* group. Fecal levels of *baiCD* were also quantified using a previously validated primer pair (Supplementary Table S2) which targets a conserved region of the gene within *Clostridium scindens VPI 12708* and *Clostridium hiranonis TO931*, two strains known to exhibit bile acid 7α-dehydroxylation activity^48, 49^.

Frozen fecal pellets were crushed into a powder and bacterial DNA was isolated using the QIAamp DNA Stool Mini Kit (Qiagen, Valencia, CA). To investigate bile acid metabolism loci, qRT-PCR was performed on a Bio-Rad CFX Connect Real-Time PCR Detection System as previously described^13^. Gene expression relative to vehicle control was calculated using the 2^−∆∆CT^ method, with each fecal sample extract normalized to total bacterial DNA levels using universal primers targeting the 16 S ribosomal RNA gene^81^. Gene levels are plotted relative to vehicle control.

### Data Availability

RNA-Seq datasets for the ileal epithelium and liver are available in the Gene Expression Omnibus (GEO; accession number GSE89430 and GSE87519, respectively). Primer sequences used for qRT-PCR are listed in Supplementary Table S2. MRM parameters used in the sugar probe permeability analysis are provided in Supplementary Table S3.

## Electronic supplementary material

Supplementary Information

Supplementary Tables
