# Supplementary material for: 2,3,7,8-Tetrachlorodibenzo-p-dioxin (TCDD)-elicited effects on bile acid homeostasis: Alterations in biosynthesis, enterohepatic circulation, and microbial metabolism
Source: Sci Rep. 2017 Jul 19;7:5921. doi: 10.1038/s41598-017-05656-8 (PMC5517430; doi:10.1038/s41598-017-05656-8)
Supplement: Supplementary file 1 — Supplementary Information [file 41598_2017_5656_MOESM1_ESM.pdf]

SUPPLEMENTARY INFORMATION

2,3,7,8-Tetrachlorodibenzo-*p*-dioxin (TCDD)-elicited effects on bile acid homeostasis: Alterations in biosynthesis, enterohepatic circulation, and microbial metabolism

Kelly A. Fader[1,2], Rance Nault[1,2], Chen Zhang[3], Kazuyoshi Kumagai[4], Jack R. Harkema[2,4], and Timothy R. Zacharewski[1,2,*]

[1]Department of Biochemistry & Molecular Biology, Michigan State University, East Lansing, MI, 48824

[2]Institute for Integrative Toxicology, Michigan State University, East Lansing, MI, 48824

[3]Department of Chemistry, Michigan State University, East Lansing, MI, 48824

[4]Department of Pathobiology & Diagnostic Investigation, Michigan State University, East Lansing, MI, 48824

*Corresponding author:
Timothy R. Zacharewski, Ph. D.
Michigan State University
603 Wilson Road, Room 309
East Lansing, MI, 48824-1319
Telephone: 517 – 355 - 1607
E-mail: tzachare@msu.edu

SUPPLEMENTARY FIGURES

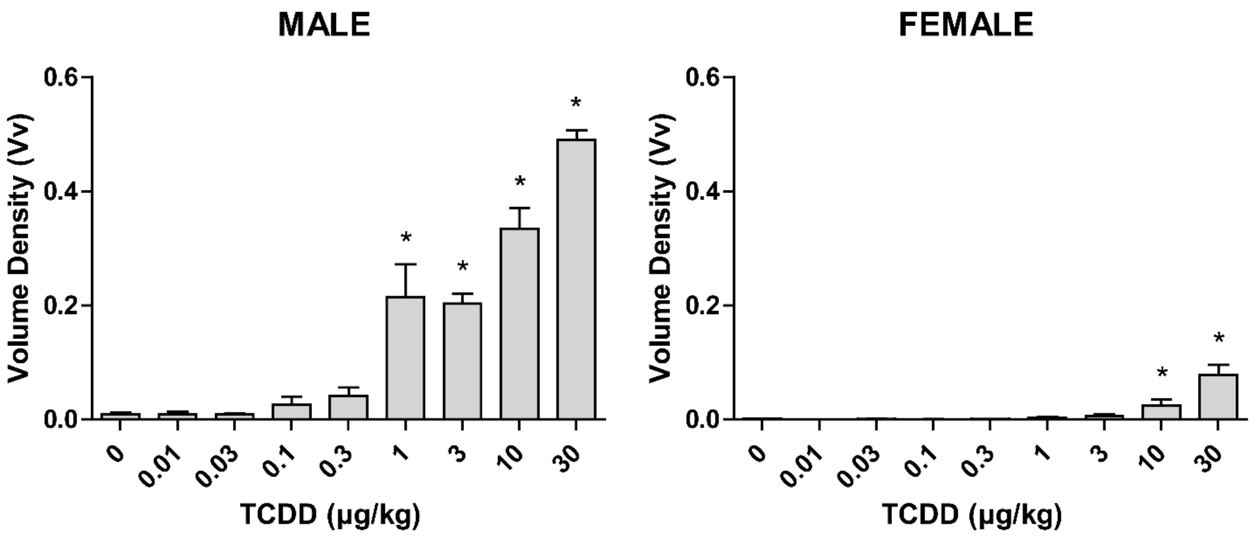

Supplementary Figure S1: Quantification of lipid accumulation in the liver of male (left) and female (right) C57BL/6 mice orally gavaged with sesame oil vehicle or 0.01-30 µg/kg TCDD every 4 days for 28 days. Lipids were stained with Oil Red O and quantified using the Quantitative Histological Analysis Tool (QuHAnT). Bars represent the average ± standard error of the mean for at least 4 biological replicates. Statistical significance (* $p \leq 0.05$) was determined using one-way ANOVA analysis followed by Dunnett's post-doc test performed in SAS 9.3.

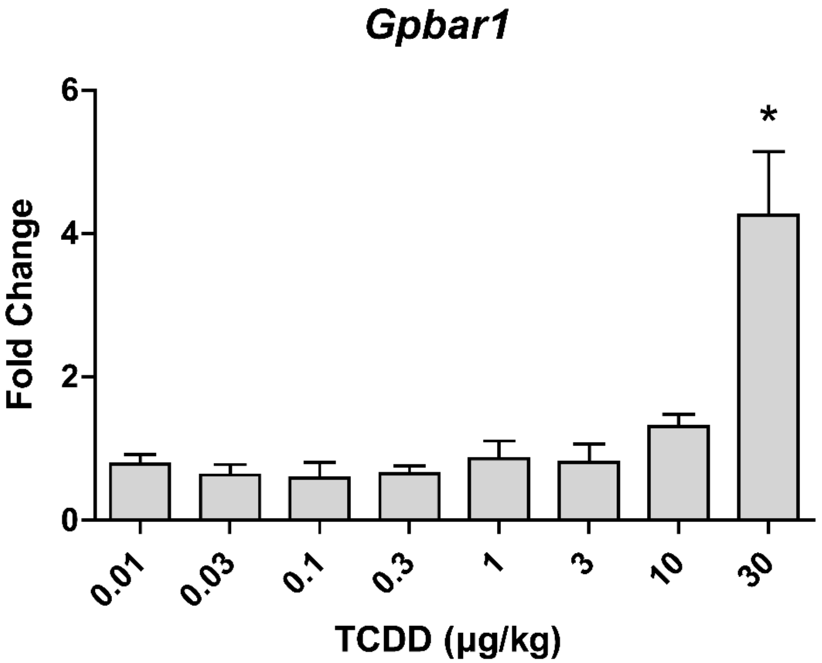

Supplementary Figure S2: Quantitative real time polymerase chain reaction (qRT-PCR) analysis of G protein-coupled bile acid receptor 1 (*Gpbar1*) expression in the liver of male C57BL/6 mice orally gavaged with sesame oil vehicle or TCDD (0.01-30 µg/kg TCDD) every 4 days for 28 days. Bars represent the average fold change relative to vehicle ± standard error of the mean (SEM) for at least 3 independent biological replicates. Statistical significance (* $p \leq 0.05$) was determined using one-way ANOVA analysis followed by Dunnett's post-doc test performed in SAS 9.3.

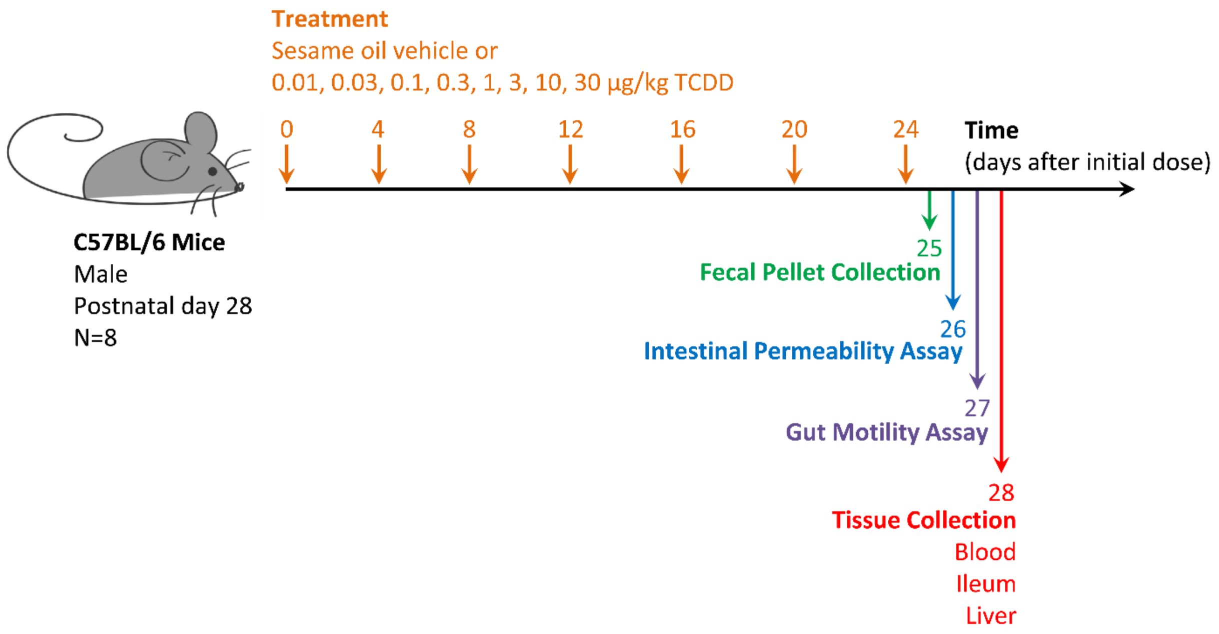

Supplementary Figure S3: Study design. Male C57BL/6 mice (n=8) were orally gavaged with sesame oil vehicle or 0.01-30 µg/kg TCDD every 4 days for 28 days.

SUPPLEMENTARY TABLES

The following supplementary materials can be found in the Excel spreadsheet titled "Supplementary Tables".

Supplementary Table S1: RNA-Seq analysis of differential gene expression in the liver and ileum of TCDD-treated mice. Fold changes (FC) represent expression relative to the vehicle control. P1(t) values represent posterior probabilities of activity on a per gene and dose basis using the model based t-value.

Supplementary Table S2A: Primer sequences (5' -3') for genes analyzed by qRT-PCR.

Supplementary Table S2B: Degenerate primer sets for bacterial genes analyzed by qRT-PCR.

Supplementary Table S3: Multiple reaction monitoring (MRM) parameters used for the tandem mass spectrometry (MS/MS) analysis of four sugar probes
